# Functional Upregulation of Ca^2+^ -Activated K^+^ Channels in the Development of Substantia Nigra Dopamine Neurons

**DOI:** 10.1371/journal.pone.0051610

**Published:** 2012-12-20

**Authors:** José A. Ramírez-Latorre

**Affiliations:** Center for Neurobiology, Columbia University, New York, New York, United States of America; University of Houston, United States of America

## Abstract

Many connections in the basal ganglia are made around birth when animals are exposed to a host of new affective, cognitive, and sensori-motor stimuli. It is thought that dopamine modulates cortico-striatal synapses that result in the strengthening of those connections that lead to desired outcomes. We propose that there must be a time before which stimuli cannot be processed into functional connections, otherwise it would imply an effective link between stimulus, response, and reward in uterus. Consistent with these ideas, we present evidence that early in development dopamine neurons are electrically immature and do not produce high-frequency firing in response to salient stimuli. We ask first, what makes dopamine neurons immature? and second, what are the implications of this immaturity for the basal ganglia? As an answer to the first question, we find that at birth the outward current is small (3nS-V), insensitive to 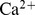, TEA, BK, and SK blockers. Rapidly after birth, the outward current increases to 15nS-V and becomes sensitive to 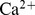, TEA, BK, and SK blockers. We make a detailed analysis of the kinetics of the components of the outward currents and produce a model for BK and SK channels that we use to reproduce the outward current, and to infer the geometrical arrangement of BK and 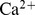 channels in clusters. In the first cluster, T-type 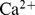 and BK channels are coupled within distances of 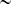
20 nm (200 Å). The second cluster consists of L-type 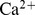 and BK channels that are spread over distances of at least 60 nm. As for the second question, we propose that early in development, the mechanism of action selection is in a “locked-in” state that would prevent dopamine neurons from reinforcing cortico-striatal synapses that do not have a functional experiential-based value.

## Introduction

The physiology of dopamine neurons of the substantia nigra (SN) has received a great deal of attention throughout the years [1] due to the role these neurons play in the regulation of the basal ganglia (BG). A classical theoretical framework that has shaped our understanding of the function of the BG is the notion that massive parallel signals that originate in the cortex are processed by the direct and indirect pathways of the BG that arise in the striatum and constitute the beginning of a formidable feedback loop that will eventually return to the cortex via the thalamus [2]–[6]. Although this classical model is very useful as a theoretical framework, it is now appreciated that the the direct-indirect model needs to be complemented with several lateral and reciprocal connections that give rise to topographically organized microcircuits that process emotional, associative, sensory, and motor information [6]. Irrespective of these theoretical considerations, it is clear that the degeneration or damage of SN DA neurons in Parkinson's disease or in animal models result in major movement alterations [2], and that some of these alterations can be attributed to the lack of DA in the striatum. It is also clear that dopamine neurons of the SN control cortico-striatal synaptic plasticity through the release of DA on striatal fields [7]–[11].

Due to the precise relationship between the synaptic input to SN DA neurons and the regulation of neuronal output through the release of DA in the striatum, it is important to understand how DA neuron excitability regulates the release of DA in the striatum. Main GABAergic input from the striatum, globus pallidus, and SNr, glutamatergic input from STN, and cholinergic input from the pedunculopontine nucleus (PPN) impinge on SN DA neurons and activate ionic mechanisms that are involved in the processing of output signals that lead to the release of DA in the striatum [12]. Using in vivo recordings, several studies concluded that SN DA neurons fire almost exclusively in a pacemaker mode, thought to control the basal DA release in the striatum [13]–[16], although more recently Blythe et al. have shown that bursting can be achieved in the slice using dendritic application of glutamatergic agonists or somatic current injection in perforated patches and whole-cell recordings [17], [18]. In vivo, as well as in some slice preparations, SN DA neurons switch between bursting and non-bursting modes [17]–[21]. Given that SN DA neurons are subject to a powerful synaptic drive, it is probable that these neurons respond with bursting to the complex interplay between excitatory synaptic input and a GABA_A_-dependent disinhibition mechanism [12], [17], [21]–[24].

Early in postnatal development however, SN DA neurons are very immature and their inability to generate trains of action potentials suggests that they would not able to sustain high levels of DA in the striatum. At birth, rat SN DA neurons produce neither trains of action potential upon depolarization, nor exhibit the pacemaker activity characteristic of mature neurons [25], [26]. The action potentials (AP) that can be produced by large depolarization are wide (5–10 ms), lack an after-hyperpolarization potential (AHP), and have an abnormal depolarization after-potential [25].

Even though single BK channels were described as early as 1981 [27], the association between BK channels and movement regulation dates back to the observation that Drosophila slowpoke (*slo*) mutants displayed abnormal locomotor behavior and less flying ability than normal flies [28]. BK channels have been implicated in the control of movement in flies, worms, mice and humans [28], [29], but only a few papers [30], have incorporated them in SN DA neuron models of excitability. It has been shown that in sympathetic and pacemaker neurons the electrical activity is regulated by interactions of Cav1.3 (L-type Ca channel) and BK channels [31], [32]. These interactions have been shown to exist in several pacemaker neurons such as suprachiasmatic neurons, adrenal chromaffin cells and heart nodal cells [32]. BK role in burst generation has been known for a long time [33] and recently Su, Song, and Li [34] have reported on the presence of BK single channels on SN DA neurons of similar characteristics to the channels we find in young SN DA neurons in the present work. Cardozo and Bean have shown that 10% of the outward current can be blocked by iberiotoxin [35] and Katayama et al. [36] have shown that mGluRs activation causes the hyperpolarization of dopamine neurons mediated by the opening of charybdotoxin-sensitive 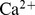 -activated 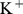 channels.

The aim of this work is the characterization of the changes that occur in excitability during development using patch-clamp recordings of SN DA neurons at different days of development encompassing the first month of post-natal life. We show that there are some components of the outward current that are not developmentally regulated, but that both components of CaAK (SK+BK) are potently upregulated in development, suggesting to us that they form part of the general developmental process by which SN DA neurons take control of the regulation of the BG.

## Results

### Developmental changes in the localization of SN DA neurons

For this study neurons were were selected initially by their size and electrophysiological properties which have been found to be good predictors of DA identity [14], [17], [37]. Of 135 neurons recorded, 29 neurons were injected with LFY and double labeled with TH. In 21 of these 29 neurons, the fluorescent image could be supperimposed with the TH staining. 5 of these 29 neurons were located in dense areas of TH staining and the image could not be uniquely superimposed with the TH staining and the rest (3) of the neurons showed no TH staining that could be identified in the area where LFY fluorescence had been observed.

**Figure 1:**
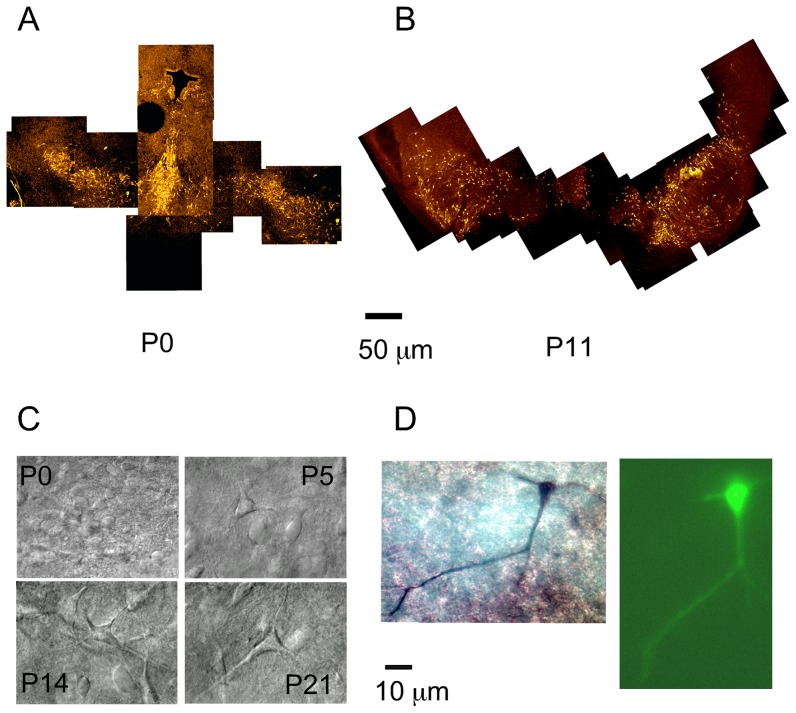
Distribution, morphology, and double staining of SN DA neurons. (A) TH staining of rat midbrain neurons in transversal sections of 200 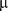
m thickness. Sections were cut, fixed, and labeled with a fluorescent anti-TH antibody as described in the methods. TH-antibody staining of a P0 rat. Migrating cells from the forth ventricle can still be observed. Bar is 50 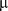
m. (B) TH-antibody staining of a P11 rat. The left-hand side SN at P11 is more rostral and shows a wide spread localization of DA neurons. The right-hand side SN at P11 is more caudal, and shows already the typical SN compacta band in the dorsal aspect, and less number of displaced dopamine neurons outside the compacta area. Dorsal is up and ventral down. (C) Infrared IR-DIC optics images of SN neurons. Rat slices prepared according to the methods section. Infrared pictures of neurons in the SN as observed during the electrophysiological recording. (D) Double stained SN DA neuron at P3. The image on the left shows the TH staining. The image on the right shows the LFY filled neuron.

Figure 1A shows the dense distribution of DA neurons in the SN at P0 (Fig. 1A, P0). As development proceeded, two processes, neuronal migration and neuronal death slowly shaped the distribution of DA neurons in the SN, restricting the location of DA neurons to the dorso-lateral and ventro-medial aspects of the SN, a territory known as the compacta area (SNc) (Figs. 1A,B) [38], [39]. By the end of the second week of development, the demarcating boundary between the compacta and the reticulata areas of the SN was apparent, but many patches with DA neurons were observed in ventral territories outside the SNc (Fig. 1B). The distribution of DA neurons depended on the rostro-caudal axis, such that the more rostral aspects of the SN have a wide distribution of DA neurons, whereas the caudal territories of the SN show a more restricted distribution of DA neurons, mostly confined to the SNc (Fig. 1B). During the third week of development, the boundary between the reticulata and the compacta areas reached its adult form. At this time, only isolated patches of DA neurons remain outside the SNc [39], [40].

Under the infrared optics used in our experiments, young neurons appeared smaller, rounder, and with less apparent processes than older neurons (Fig. 1C). Figure 1D illustrates the procedure used to identify DA neurons. After the experiment, the patch was ruptured with suction and Lucifer Yellow (LFY) and biocytin were allowed to diffuse into the cell for 3 minutes. Figure 1D left shows the TH staining, and the right panel in figure 1D shows the LFY fluorescence. Both images were superimposed thus allowing a positive identification of the recorded neuron as a TH positive neuron.

### Changes in electrical properties of SN DA neurons with development

**Figure 2:**
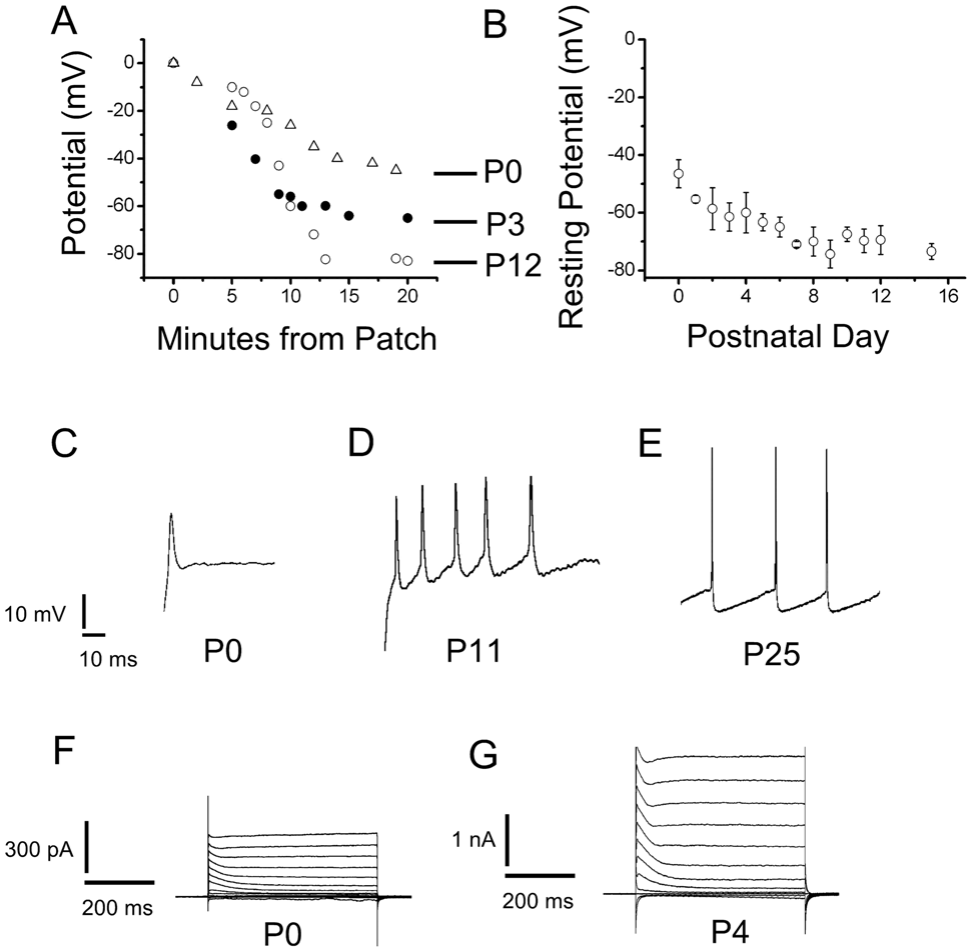
Excitability Changes in SN DA neurons during development. (A) Resting potential measurements for three SN DA neurons as a function of time. After obtaining a cell-attached patch, we waited to gain access as the gramicidin diffused from the back of the pipette onto the tip, measuring the voltage drop necessary to maintain a zero current, in the search mode. The resting potential equilibrated slowly as gramicidin was incorporated into the patch. Only recordings that developed over the course of several minutes and were stable after reaching equilibrium were used. Open triangles, neuron at P0. Closed circle, neuron at P3. Open circles, neuron at P12. (B) Resting potentials measured with the method illustrated in A, are plotted as a function of postnatal day of development. (C),(D) & (E) Electrical activity of SN DA neurons in response to depolarizing current pulses. Neurons in the fast-current clamp mode, were depolarized by current injections. P0 to P5 neurons never displayed spontaneous firing. The P0 neuron shown here was depolarized to −20 mV, from a resting potential of −40 mV. The P11 neuron was depolarized to −40 mV from a resting voltage of −60 mV. The P25 could fire a continuous train of action potentials from resting potential of −60 mV. Small current injections were performed to adjust the resting voltage. Small current injections were performed from resting voltages (Notice difference between P0 and P11). This was on purpose to avoid large current injections that could distort the recording. However, even when depolarized from −60 mV, P0 and P1 neurons never fired action potential continuously, but rather the small wide action potentials shown here. (F) Typical voltage clamp current in responses to voltage pulses with 20 mV increments from a holding potential of −80 mV to test pulses from −120 to 100 mV with 20 mV increments. Raw traces shown, no leak subtraction. P0 neuron shows the typical outward current with an inactivating ***I_A_*** current and a non-inactivating current. Small ***I***_h_ and inward rectifier currents were also observed at hyperpolarized potentials less than −80 mV. (G) A P4 neuron with a larger outward current, in response to the same voltage protocol as in F. This neuron also had larger ***I***_h_ and inward rectifier currents, not analyzed here.

During the first week of postnatal development, SN DA neurons had resting potentials that were typically in the range −40 to −60 mV (Figs. 2A and 2B). Figure 2A shows a perforated patch recording in which the pipette potential was allowed to drift in the slow current-clamp (search) mode. The incorporation of more gramicidin channels into the membrane slowly equilibrated at the resting potential. The use of perforated-patches was important to avoid excessive runoff especially of 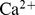 -activated 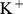 channels, an effect compounded by the use of 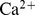 buffers in mechanically-ruptured whole-cell recordings. Three typical recordings are shown here. Figure 2B shows how resting potentials gradually shifted to the −60 to −80 mV range during the second week of development. During the first two weeks of development, neurons seldom displayed the pattern of regular firing generally observed in older animals. Figures 2C, D, and E, show some typical firing patterns of SN DA neurons at different points of development (P0, P10 and P24). The spikes observed during the first day of development (P0, Fig. 2C) were broad (5
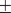
0.8 ms, n = 5), had a low threshold (V 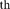 = −17
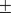
5.2 mV, n = 5) from a holding potential, (V_h_ = −60 mV), and small amplitude (V 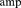 = 30
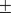
5.2 mV, n = 5, Fig. 2C). With development, the spike width decreased (2
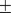
0.25 ms, P20–P25, n = 7, Fig. 2E), the firing threshold shifted towards more hyperpolarized voltages (V 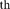 = −40
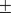
4.1 mV, P20–25, n = 7, Fig. 2E, V_h_ = −60), and the amplitude of the action potential (AP) increased (V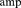 = 55
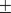
4.3 mV, P20–P25, n = 7) (Fig. 2 C–E). P0 and P1 neurons never fired action potential continuously, but rather the small wide action potentials shown in fig. 2C even when depolarized from manually clamped negative voltages, such as −60 mV.

### Developmental profile of the outward current

**Figure 3:**
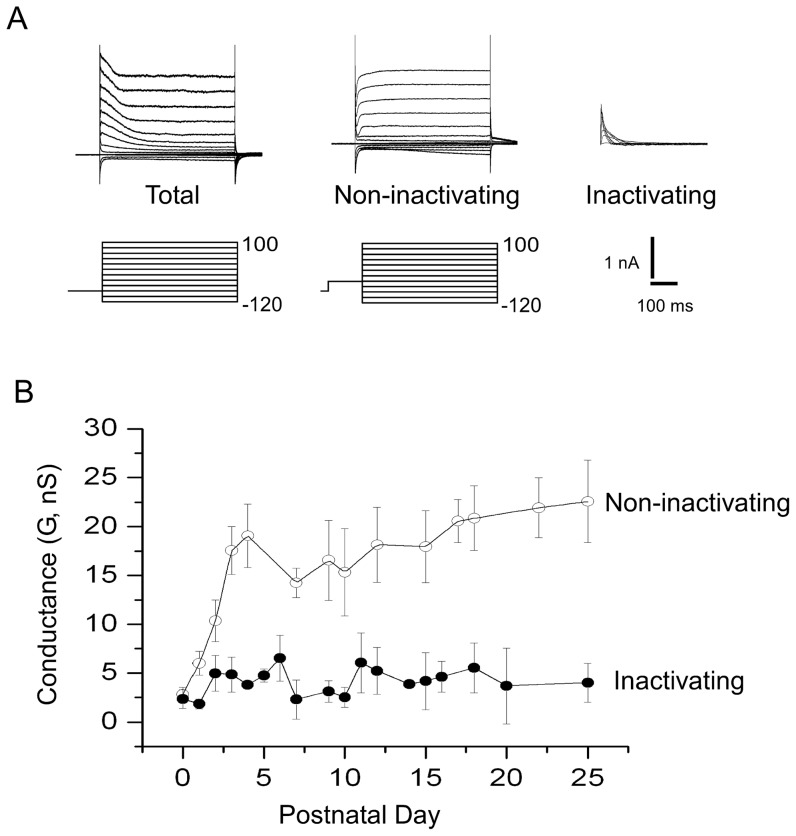
Developmental profile of the components of the outward current. (A) Left, I–V currents obtained with the following voltage protocol: currents in response to test pulses from −120 to 100 mV with increments of 20 mV, from a resting potential of −80 mV. (A), middle, same protocol as in A-left, except that a pre-pulse to −40 mV was used, leaving only the non-inactivating component. (A), right. ***I_A_*** currents were obtained by subtraction. (B) The non-inactivating and inactivating components of the outward currents were measured as a function of postnatal day of development. The conductance was assayed at 100 mV where both components of the non-inactivating outward current are fully activated.

Figures 2F and 2G show two typical perforated whole-cell current recordings of SN DA neurons at P0 and P4. During this period, there was a marked increase in the magnitude of the outward current in these neurons (Notice the difference in scale). The outward current could be broadly divided into two main components: the first component was a fast activating and inactivating 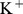 current blocked by 4 mM 4-AP (***I_A_***-type). The second component was a non-inactivating current. We measured the developmental changes of the non-inactivating and inactivating components of the outward current, using the voltage pre-pulse shown in figure 3A to subtract the inactivating component from the total outward current. I–V currents were obtained with the following voltage protocol: currents in response to test pulses from −120 mV to 100 mV with increments of 20 mV, from a resting potential of −80 mV (Fig. 3A left). The non-inactivating component was obtained when the voltage pre-pulse (−40 mV) inactivates the ***I_A_*** component (Fig. 3A middle). ***I_A_*** currents were obtained by subtraction of the non-inactivating component from the total outward current (Fig. 3A right). Figure 3B shows the evolution of the total non-inactivating and inactivating component of the outward current as a function of postnatal day. Fig. 3B shows a dramatic increase in the non-inactivating component of the outward current while the inactivating component shows no developmental regulation. The change in the non-inactivating current was about 4–5 fold higher than at P0 after the first week of development, while the changes in the inactivating current are statistically insignificant from those at P0.

### Developmental changes in the sensitivity of the outward current to TEA

**Figure 4:**
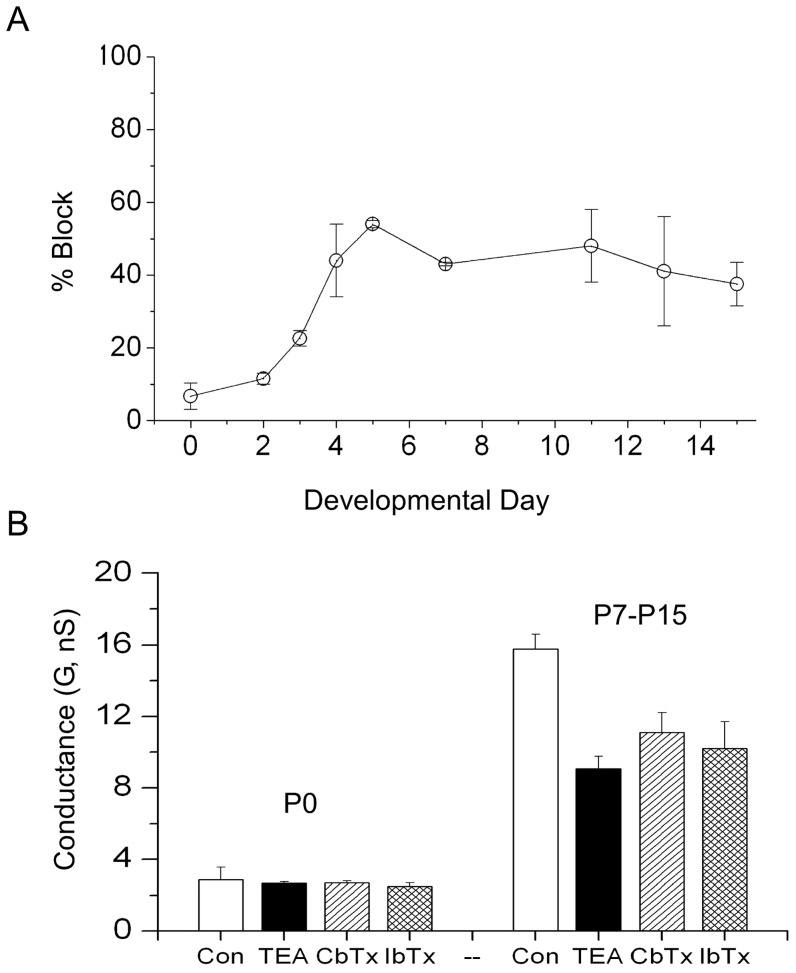
TEA sensitivity of the outward current as a function of postnatal day. (A) The reduction of the outward current to 20 mM TEA was assayed with the protocol in figure 7. (B) Comparison of the differences in pharmacology for the total non-inactivating outward current, between early (P0) and late (P7–P15) SN DA neurons. Experimental protocol as explained in figure legends 5 & 6. Control conductance (Left white bar, G(Control, P0)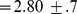 nS, n = 10). At P0 the currents are insensitive to 20 mM TEA (Left Black bar, G(TEA, P0)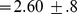 nS, n = 4), 1 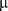
m CbTx (Left hatch bar, G(CbTx, P0)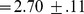 nS, n = 3) and 1 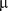
m IbTx (left criss-cross bar, G(IbTx, P0)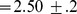 nS, n = 2). Average control conductance pooled from P7 to P15 (Right white bar, G(Control, P7–P15)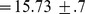 nS, n = 23). Effect of 20 mM TEA on the conductance from P7–P15 (Right black bar (G(TEA, P7–P15)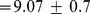 nS, n = 10). Effects of 1 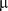
m CbTx (Right hatch bar G(CbTx, P7–P15)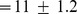 nS, n = 3) and 1 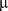
M IbTx (Right criss-cross bar, G(IbTx)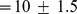 nS, n = 2) on the conductance, data pooled from P7 to P15.

We determined the changes in the percentage block of 20 mM TEA on the outward current as a function of development. The results are shown in figure 4A. The current at P0 was insensitive to TEA (6.7%
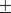
3.7), while typically 40% the outward current was blocked by TEA during the second week of development. Figure 4B shows that the P0 current was also insensitive to IbTx and CbTx, while the data pooled from several experiments at different developmental ages suggest that some of the TEA current can be blocked with IbTx and CbTx. These data suggest an involvement of BK channels in at least a good portion of the outward current.

### Early outward 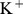 currents are TEA insensitive (*I* 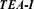) and weakly voltage dependent

**Figure 5:**
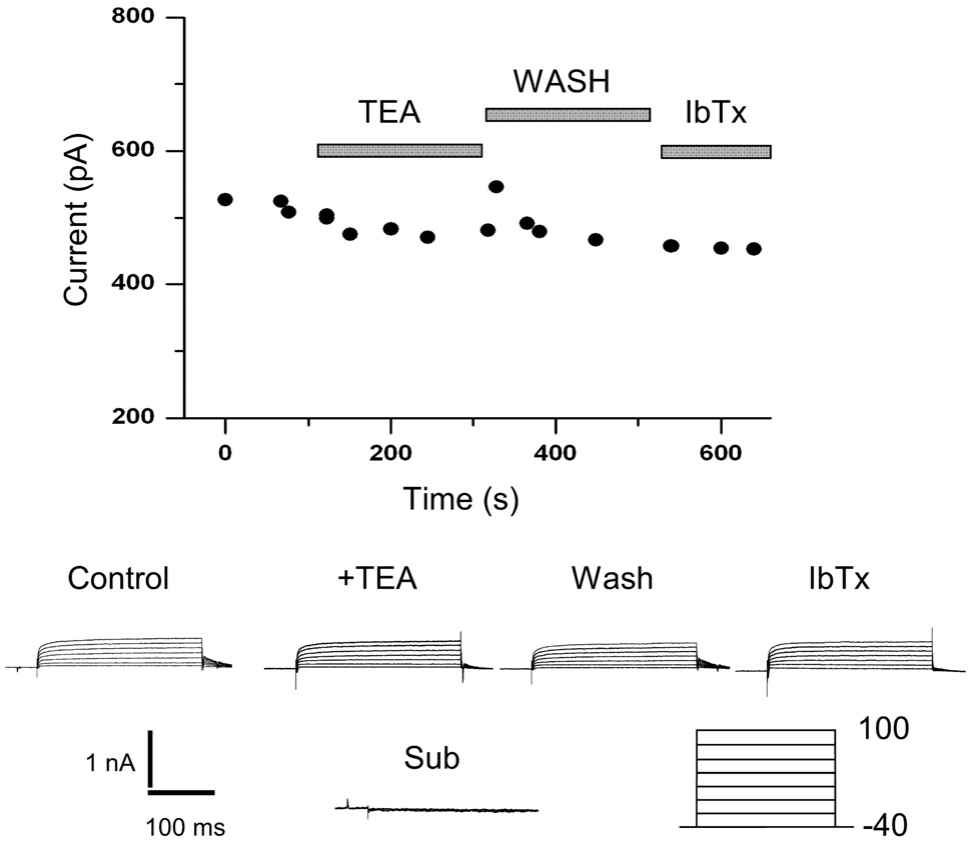
P0 outward currents are insensitive to TEA. Perforated whole-cell current of a SN DA neuron at P0. The whole cell patch was obtained and the current was recorded for five minutes to assay for decay. After 5 min, 20 mM TEA was applied in a fast perfusion system. No appreciable diminution of the current was observed. The slice was then washed. After the wash, 1 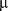
M IbTx was applied. No effect was observed on the outward current. The voltage protocol is indicated below the trace.

The non-inactivating component of the outward current has two sub-components: a TEA insensitive sub-component (***I*** 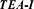), and a TEA sensitive sub-component (***I*** 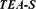) blocked by 20 mM TEA. At P0, the outward current is made almost completely of the TEA insensitive current and its activation is slow and voltage independent. Figure 5 shows an experiment that demonstrates the insensitivity of the P0 outward current to application of TEA (20 mM) and IbTx (1 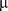
M). The kinetics of activation of this P0 current can be fit by an exponential of the form 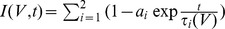. This TEA insensitive current activated with a fast time constant of 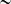
4–5 ms for all voltages shown (Pulses from −40 to 100, +20 mV steps) and a slow time constant of 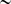
85 ms. The activation of the P0 current can be fit with a 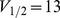 mV and 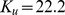 mV. The ***I*** 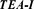 current at P0 is also insensitive to application of 1 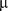
M Charybdotoxin (CbTx) and 1 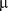
M Iberiotoxin (IbTx) (See Fig. 4).

### The TEA sensitive (*I* 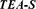) component of the outward current

**Figure 6:**
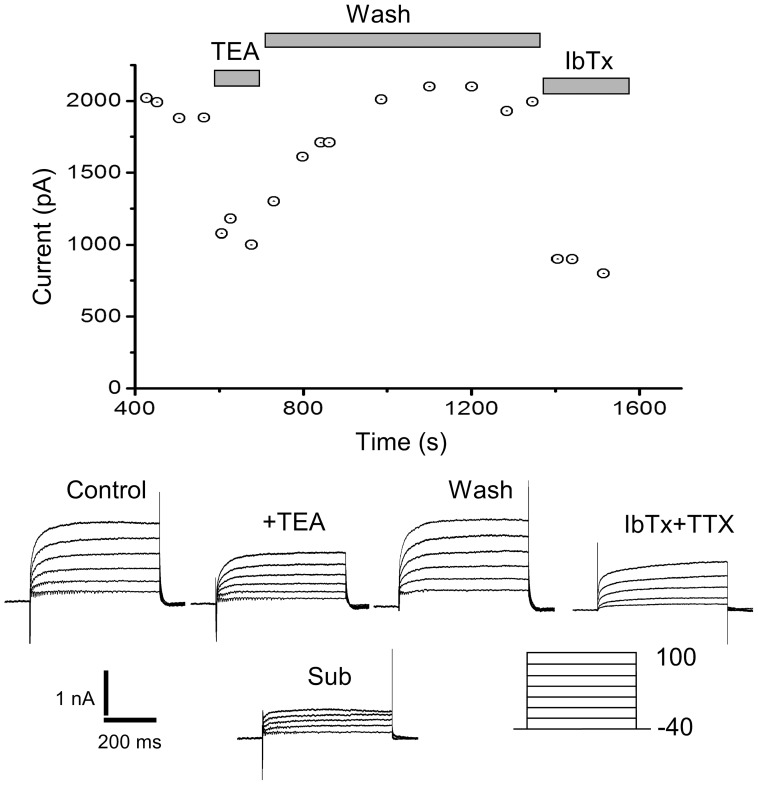
The TEA sensitivity of non-inactivating outward currents. A perforated whole-cell patch on a P7 neuron is sensitive to TEA and IbTx. Voltage protocol similar to the one in figure 5, indicated below the traces. The currents were monitored for 5 minutes before the perfusion of TEA (Control trace). Fast perfusion with 20 mM TEA causes a fast reversible block of the current (Traces marked + TEA). A wash slowly recovers the current to near control values (Marked wash). Application of IbTx (1 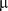
M) causes an irreversible block of the current, similar to the block caused by TEA.

The second sub-component of the non-activating component of the outward current is a TEA sensitive subclass (***I*** 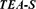) that rapidly develops after P0. Figure 6 illustrates the experimental protocol used to isolate this component in a P7 neuron. After whole-cell access was achieved, we recorded for 5 min. to assess the rundown of the current. In figure 6, the outward current was stable and the rapid application of 20 mM TEA caused a fast and reversible reduction of the non-inactivating outward current. In most cases, TEA reduced the current within seconds of its application. The reduction of the current could be slowly reversed after removal of the TEA, using ringer solution to wash the slice (Fig. 6). At the end of this wash, we applied IbTx (1 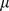
M). IbTx blocked the fast components of the outward current. Kinetic analysis of the current blocked by IbTx, indicated a close similarity to the current blocked by TEA. In figure 6, the TEA sensitive (***I*** 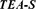) current was obtained by subtracting the TEA insensitive current (***I*** 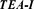) from the outward current before the application of TEA.

### Kinetics of the *I* 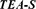 and *I* 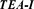 currents

**Figure 7:**
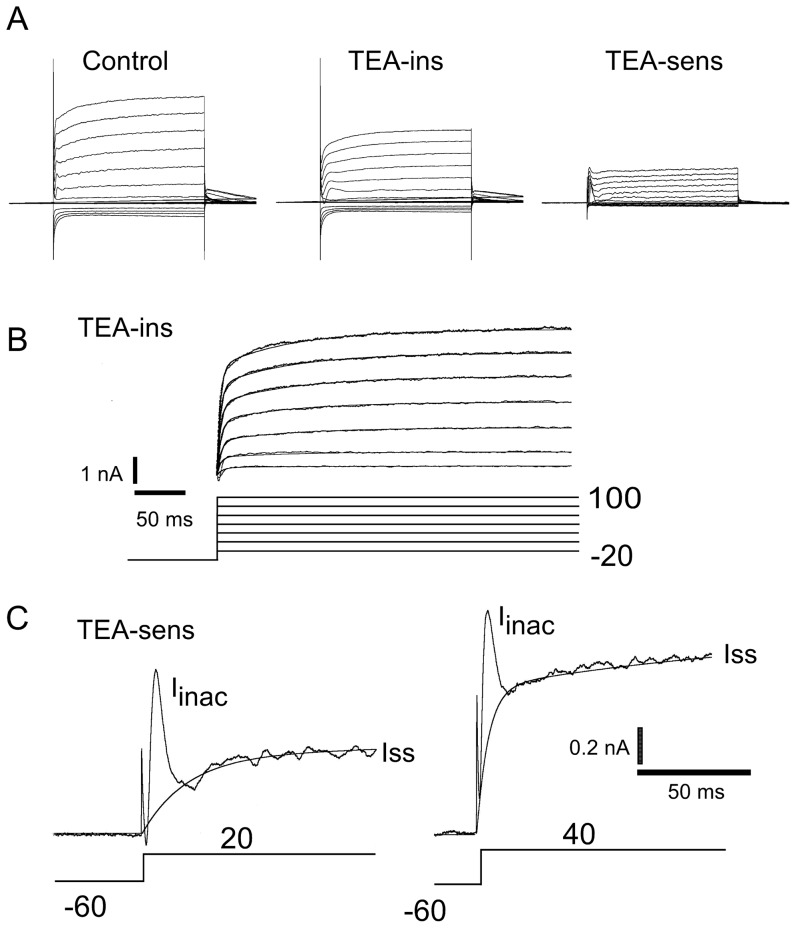
Detailed analysis of the kinetics of the TEA sensitive current. (A) Perforated whole-cell patch on a P15 SN DA neuron. Currents are in points, fits are continuous lines. Protocol identical to the one used in figure 6. Control trace (marked CONTROL) is shown before the application of 20 mM TEA. (B) The application of TEA reduces the current to the TEA insensitive current (***I*** 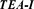). The trace shown is obtained from panel A middle after a capacitance subtraction. This TEA-insensitive current has the characteristic 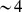 ms activation seen in figures 5 at P0. This current was fit according to the equation 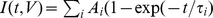. This fit is superimposed on the whole-cell current as a continuous line. Two time constants were necessary to fit the data, a voltage independent fast component of 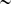
4–7 ms, and a slow one of 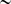
85 ms (V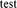 from −20 to 100 mV, step 20 mV, the slow component was fit by 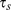 = 85, 85, 85, 86, 88, 84, and 85 ms respectively). (C) Two components can be detected in the ***I*** 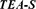 current of the SN DA neuron. A step depolarization to +20 mV (C left), or +40 mV (C right) produces two types of currents that are sensitive to TEA. The ***I*** 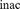 component is characterized by a voltage dependent activation and a voltage independent inactivation time constant (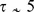 ms). The ***I*** 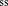 component is characterized by a voltage dependent activation time constant. The lines are the fits to the ***I*** 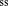 current shown. In the left trace, 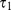
(activation)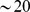 ms, and in the right hand side, 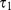
(activation)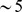 ms. A second voltage dependent component with slower time constants was used to fit these data (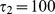 ms, lhs panel and 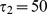 ms, rhs panel).

Figure 7 shows an expanded version of a TEA blocking experiment. Increasing the pre-pulse to −60 mV brings out another component of the outward current, a fast inactivating small component, seen at the beginning of the traces. Panel A shows a typical trace before (CONTROL) and after (***I*** 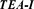) the application of 20 mM TEA. The trace labeled TEA-sens is the result of subtracting the trace after the application of TEA (***I*** 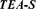) from the control (CONTROL) trace. Figure 7B shows the the residual current left after the application of TEA (***I*** 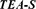). The fit shown here was activated with a slow time constant (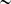
4–7 ms) and an activation function characteristic of the outward current at P0 (See Figs. 5 & 6 for comparison). Figure 7C shows an expanded view of two traces of the TEA-sensitive component shown figure 7A (panel 7A, right) where two components of the ***I*** 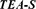 can be easily identified, ***I*** 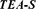 = ***I*** 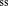 **+** ***I*** 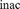 (Fig. 7C). The ***I*** 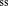 component is a voltage dependent non-inactivating current. The ***I*** 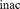 component is characterized by a fast voltage-dependent activation and a voltage-independent (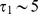 ms) time constant of inactivation. For the traces of figure 7C, the activation time constants for Iss were 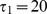 ms (Left panel), and 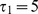 ms (Right panel). A second voltage-dependent component with slower time constants was used to fit these traces (Fig. 7C, 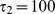 ms, left, and and 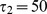 ms, right).

### The *I_A_* subclass: biophysical properties

**Figure 8:**
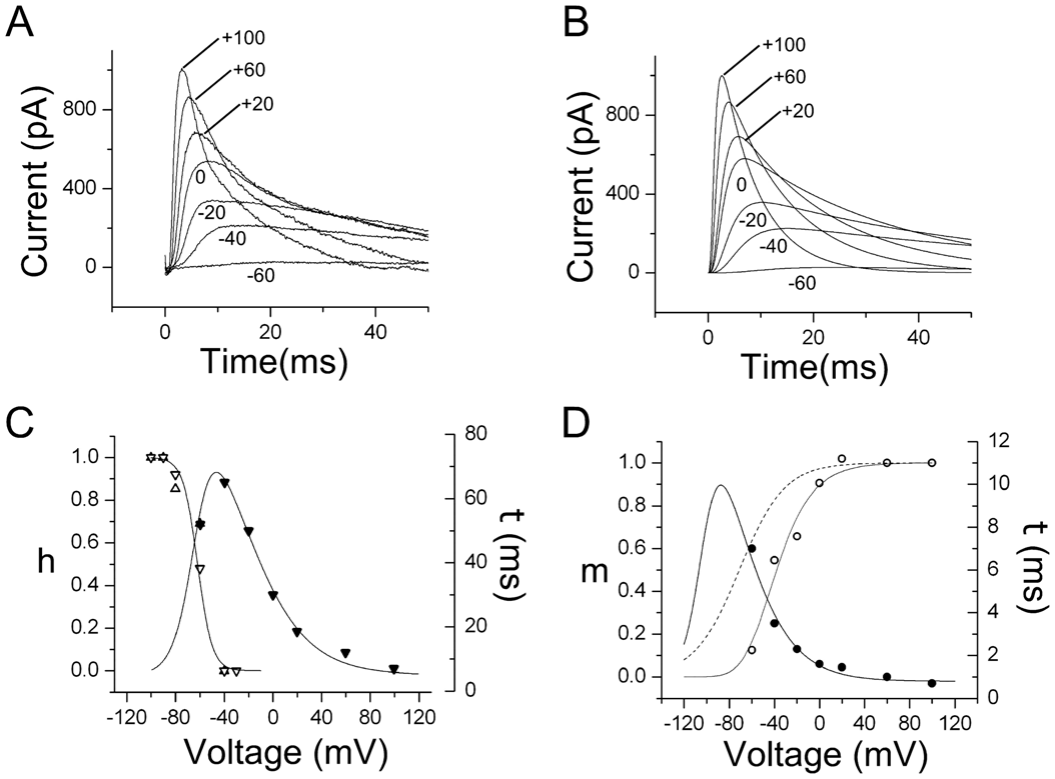
Biophysical characteristics of the *I_A_* current. A perforated patch on a P3 SN neuron that was later identified as a DA neuron. (A) I–V currents obtained with the same voltage-pulse protocol used in figure 3A. A small correction was used to account for a small difference in leak current. Voltage as indicated, the traces at 40 and 80 mV were excluded for clarity. (B) Fit to the currents on A was performed using a voltage-clamp simulator and solving Hodgkin-Huxley equations. (C) Inactivation parameters as a function of voltage. Open triangles, inactivation function, left ordinate. Closed triangles, time constant, right ordinate. The inactivation form was fit with a function of the form, 
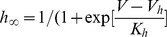
, with 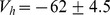, and 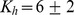 (units in mV). The function to fit the time constant was 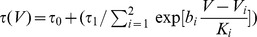 with 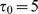 ms, 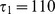 ms, 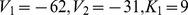, and 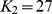 (units in mV), 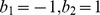. This function is shown as the fit to the closed circles with right ordinate. Identical fits were carried out at P0, P2 and P5, with similar results for activation and inactivation parameters. For P0, the activation and inactivation parameters were 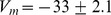, and 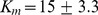 (Activation) and 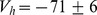, and 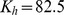 (Inactivation). (D) Activation parameters as a function of voltage. Open circles, activation function, left ordinate. Closed circles, time constant, right ordinate. The m^4^ activation data was fit to the function 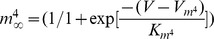, with values (mV), 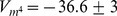, and 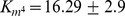. This function is shown as the fit to the open circles with left ordinate. The m activation is shown as a dashed line with the following parameters, 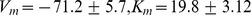 (units in mV). The time constant was fit with the same functional form as the one used for inactivation, with 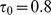 ms, 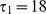 ms, 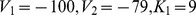, and 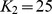 (units in mV), 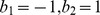. This function is show as a fit to the closed circles with right ordinate.

We investigated the biophysical properties of the ***I_A_*** current present in SN DA neurons. Figure 8A shows one such current in a P3 SN neuron, later identified as a DA positive neuron. Using the same pulse protocol as in figure 3A, the ***I_A_*** current was obtained as shown in figure 8A (40 and 80 mV omitted for clarity). Figure 8B shows the fit to the currents in figure 8A obtained by solving the Hodgkin-Huxley [33] equations and the fitting activation and inactivation parameters to approximate the simulated current responses to the currents obtained in figure 8A. Figures 8C&D show the inactivation and activation functions and time constants used to generate the ***I_A_*** currents in figure 8B. The functions used for the fit were of the type 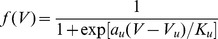, such that 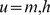, where m represents activation and h inactivation. For inactivation 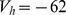 mV, 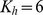 mV and 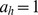 (Fig. 8C). The values used for the m^4^ activation fit are 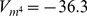 mV, 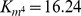 mV and 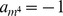 (Fig. 8D). The fit of the m activation is shown as a dashed line in figure 8D and was generated using 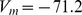 mV, and 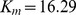 mV. The time constants were fit with the exponential functions described in figure legend 8. No statistically significant changes in the activation and inactivation parameters were observed during first two weeks of postnatal development. At P0, for example, the activation and inactivation parameters were 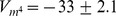 mV, and 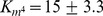 mV (m^4^ activation) and 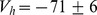 mV, and 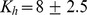 mV (h inactivation, see Fig. 8 legend).

### Other cation currents in SN DA neurons

Most of the neurons that we recorded from showed an ***I_h_*** (hyperpolarized-activated current) both in the cell-attached and the whole-cell configuration. A small inward 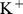 current was measured at hyperpolarized potentials, probably corresponding to an **IRC** type 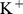 current, but the characterization of this current was not pursued in this paper.

### Modulation of the outward current by extracellular 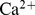


**Figure 9:**
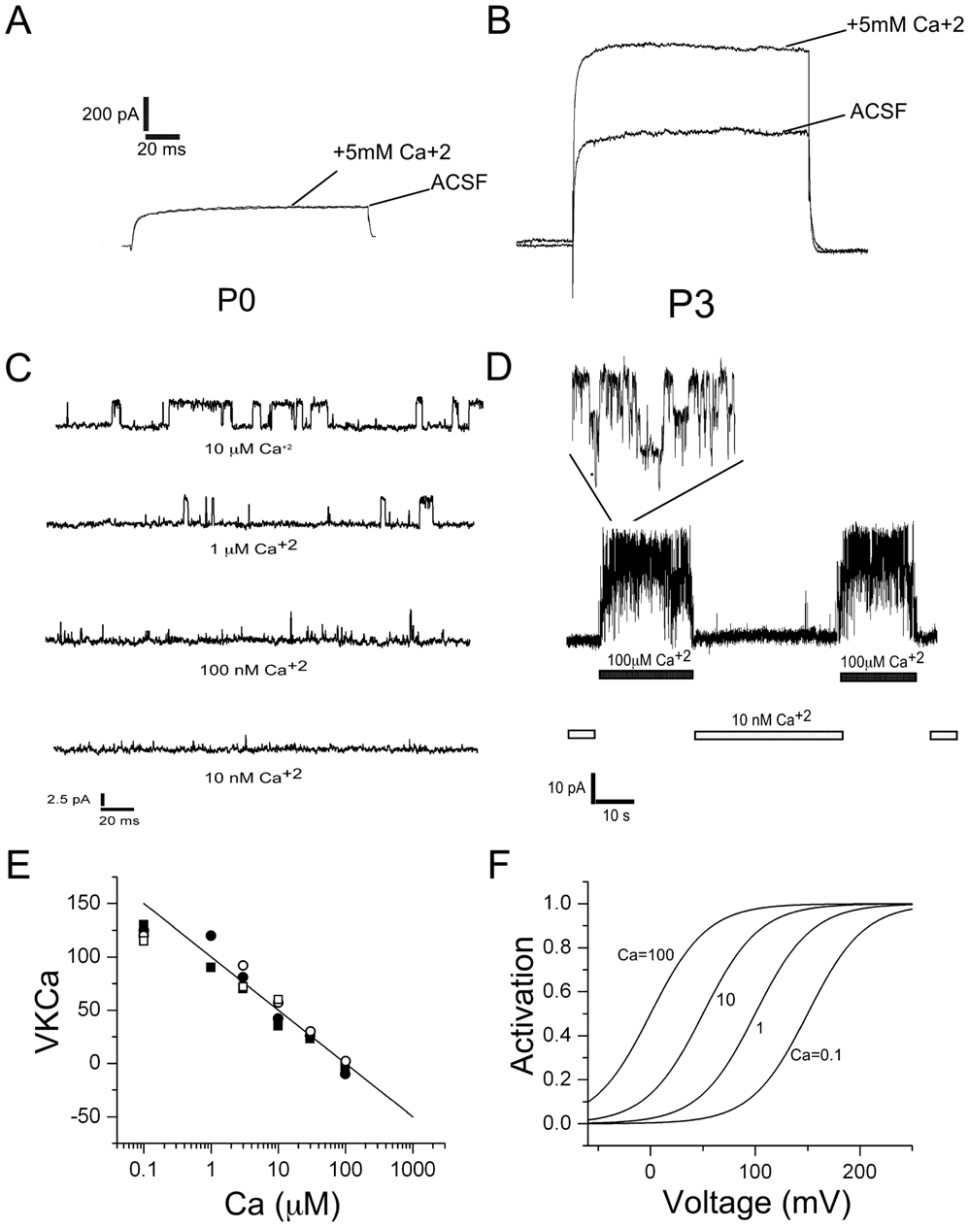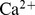 sensitivity of the outward current and single-channel recordings. (A) Outward current of a P0 neuron in regular ACSF (no calcium) and in ACSF +5 mM CaCl_2_. (B) Outward current of a P3 neuron in regular ACSF (no calcium) and in ACSF +5 mM CaCl_2_. *CaAK channels activated by intracellular* 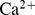. Pipette solution as in the methods. (C) Single channel recordings on a P3 SN DA neuron at voltage recording = 50 mV and 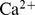 of (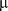
M): 10, 1, 0.1 and 0 (nominal zero). Single channel conductance 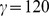 pS. (D) An excised inside-out patch obtained from a P3 SN DA neurons shows the presence of BK channels activated by internal 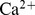 concentration (100 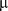
M). V = 100 mV, 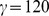 pS. (E) Half-activation function of BK channels were obtained from single channel records such as in C. The half-point activation points were measured as a function of calcium. Open circles, P3, closed circles, P7. Closed diamond P14. Closed squares, P17. The solid fit represents the equation 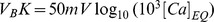, where [Ca]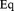 is 0.0001 mM. (F) Model of the activation function of BK channels as a function of voltage and calcium. The shift in the activation function were obtained from the mid point of the open channel probability and were fit using the function V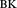, where [Ca]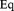 is the equilibrium intracellular Ca concentration in mM (0.0001) and [Ca]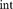 is the concentration at the mouth of the pore. For the curve labeled d, [Ca]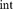 = [Ca]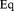 and V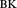 = 150 mV. For c, [Ca]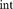 = 10[Ca]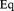 and V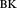 = 150 mV. For b, [Ca]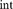 = 100[Ca]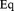 and V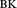 = 150 mV and for a, [Ca]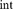 = 1000[Ca]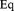 and V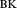 = 150 mV. These curves give the activation constant as a function of voltage and the calcium concentration in the vicinity of the channel according to equation (1), where K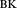 is 27 mV.

In older animals the outward current was always potently modulated by changes in extracellular 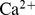. This sensitivity, however, was developmentally regulated as shown in figure 9, where the 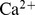 sensitivity in SN DA neurons is contrasted between P0 and P3. The outward current is not sensitive to increases in extracellular 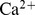 at P0 (5 mM 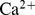, Fig. 9A), but at P3 the outward current was powerfully modulated by increases in extracellular 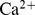 (5 mM 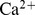, Fig. 9B).

### Single calcium-activated 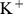 channels

Single channel experiments on excised inside-out patches on SN DA neurons revealed the presence of single channels with large single-channel conductances that are sensitive to micromolar 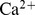 (Fig. 9C, D). These channels have conductances in the range 120–150 pS (Fig. 9C, D). The presence of BK channels on SN DA neurons has been recently reported [34]. The single channel conductance we report here is lower than that reported by Su et al. [34], but several differences between experimental conditions can explain this difference: their 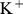 concentration was higher (140 mM vs 120 mM here) and their Mg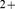 concentration in the pipette was lower than in our experiments (0.2 vs 2.0 mM here). We also substituted most of the Cl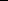 by gluconate, which in our hands shifts the activation function of the channel. Additional experiments indicate that these channels are not present at P0, while 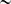
33% of the excised patches examined between P3 and P7 contain these BK channels sensitive to IbTx (1
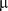
M) (No channels observed at P0, n = 10, while 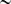
30% of the patches between P7 and P10 had these channels, n = 27). We observed that BK channels near somatic sites that gave rise to dendrites tended to be clustered such as those shown in figure 9D, whereas BK channels far from these sites were not arranged in clusters (Fig. 9D). Panel 9E shows the midpoint of the open probability as a function of voltage estimated from single-channel open probability (Po) data. Panel 9F represents the model used for the BK channel probability of opening based on the shift of the open probability curve as a function of 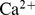.

### Mathematical model of BK currents

We modeled the BK currents using activation and time constants as functions of voltage and calcium. This model should be able to reproduce the macroscopic currents that we observe. We also wanted to use our model to estimate the proximity of the BK channels to points of 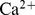 entry. The steady state activation function and time constants were fit with the following equations
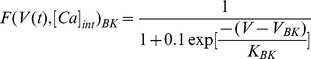
(1)and,
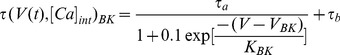
(2)where 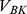 is the half activation point which is dependent on the 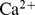 concentration sensed by the BK channels, and 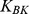 is 27 mV. From our single channel recordings (Fig. 9 C & E and legend), we have fit 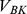 to the half-point channel activation of open times shown in figure 9E (line) as the function 
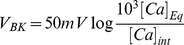
, where 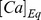 is the equilibrium calcium concentration (0.1 mM) and 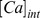 is the concentration at the mouth of the pore. With these numbers, we get an approximate 50 mV per decade change in the calcium concentration. For example, for 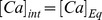, 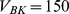 mV (Figure 9F, labeled Ca = 0.1); for 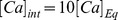, 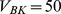 mV and for 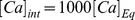, 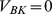 mV (Fig. 9 E&F). and 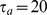 ms, 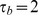 ms.

**Figure 10:**
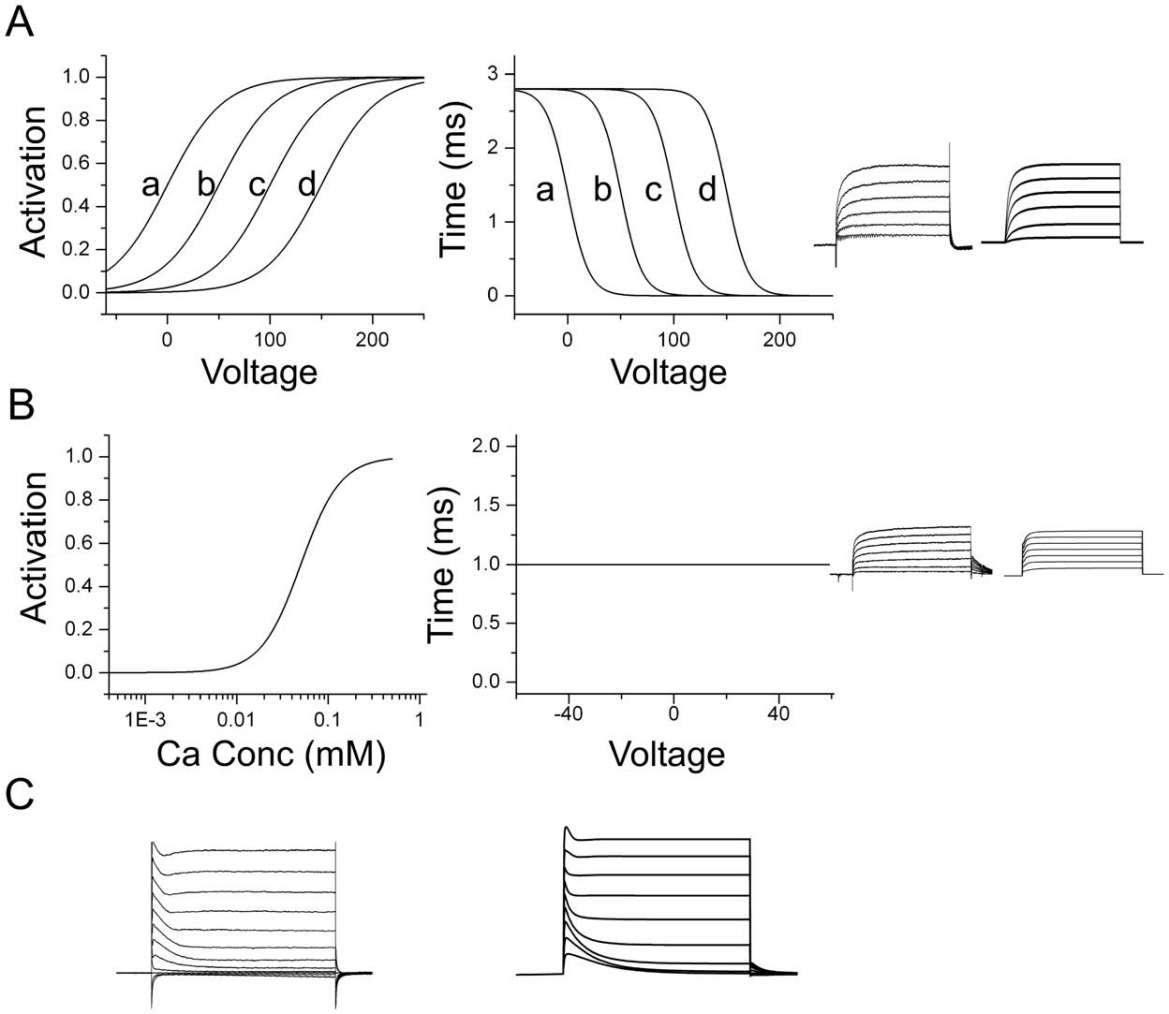
Modeling of BK and SK currents in SN DA neurons. (A). Model of the BK current in SN DA neurons. Left Panel. Activation functions were obtained as in figure 9F, shown here for clarity. Activation function and time constant of the BK currents fit to the rising phase of the experimental currents were fit using eqs. (1) and (2) with 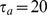 ms, 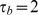 ms and V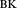 and K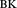 as defined in figure 9F. Right Panel. BK currents generated using the MATHEMATICA NDSolve function. The calcium concentration was solved in parallel for this currents using a procedure similar to the one described in figure 11. (B) *Model of the SK current in SN DA neurons*. Left Panel. Activation function estimated from the fit to experimental currents using Eq.(4), where K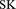 = 0.01 mM. Middle panel. Time constant of activation for SK currents were directly derived from the fit to the experimental currents. This time constant is voltage-independent consistent with the voltage-independent activation postulated for this current. 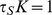 ms. Right Panel. SK currents generated using a MATHEMATICA protocol similar to the one described in figure 11. (C) Left panel. Currents obtained from a P4 neuron in response to depolarizing voltage pulses (Same as in figure 3A, left). TTX used to block 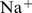 channels. Right panel. Model neuron incorporating the ***I_A_***, ***BK***, and ***SK*** currents in response to depolarizing voltage pulses. No attempt made at fitting exactly the curves and this panel shows in a qualitative sense how the model approximates a typical outward current recording from a SN DA neuron.

Equations 1 and 2 were used to solve the following equation for the time dependent activation function
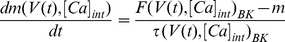
(3)which is a generalized Hodgkin-Huxley equation; figure 10A shows a summary of the model generated for BK channels. Panel A left shows the BK activation functions (same as panel 9F, shown here for clarity), panel A center shows the time constants as a function of voltage and calcium, the letters a, b, c, and d indicate 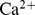 concentrations equal to: d) 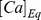 (0.1 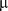
M), c) 10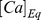, b)100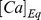 and a) 1000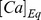 (100 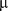
M). Panel A middle shows the BK time constants as function of voltage and 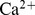 concentration where the letters a,b,c, and d indicate same 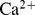 concentrations as in panel A left. Panel A right shows a comparison of an experimental BK current with the model. 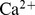 currents were generated with an HVA-L-type channel described in the next section.

### Model of SK currents

The current through SK channels was modeled using an activation constant that is independent of voltage, but dependent on the calcium concentration. The activation function is of the form
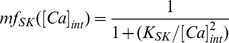
(4)and the time constant, 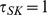 ms. Figure 10B shows the activation function(left panel), time constant (center panel), and the experimental currents obtained to depolarizing pulses and the model (right panel).

### Comparison between experimental outward current and model

Figure 10C right shows the total outward current that we obtain with our simulation when we include the ***I***_A_, ***BK*** and ***SK*** components of the outward currents, and compare them with the experimental outward current (Fig. 10C right panel). No attempt was made to fit the experimental currents exactly, but rather from the fit to individual currents, only the magnitude of the conductance was adjusted and in figure 10C, the experimental curves and the model are shown for qualitative comparison. The CaAK components, SK and BK, are very important part of the outward current and they are both essential to approximate the total outward current observed under experimental conditions.

### Modeling of inactivating BK current

In perforated whole-cell recordings, the majority of cells have a fast activating and fast inactivating BK current (***I*** 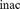, fig. 7C). This current is specifically sensitive to BK blockers and toxins and is subject to run-down if the patch is ruptured, indicating that patch pipette 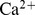 -buffering may interfere with its normal activation. Inactivating BK currents have been observed in chromaffin cells [41], hair cells [42], and in neurons of the hyppocampus [43], amygdala [44], and neocortex [45]. Although it is appreciated that some mechanisms of desensitization may depend on the co-expression of 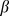 subunits with the 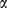 subunit that forms the pore [46], [47], a BK channel in close proximity with a inactivating 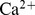 source would produce BK inactivation. This is the mechanism with which we modeled the inactivating BK current (***I*** 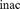, Fig. 7C), and we used the available data on 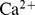 channels known to be present on SN DA neurons. Among these 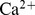 channels, the L-type appears to be important for firing [35], [48], [49], but the role of other HVA types in modulation of firing has not been ruled out [49]. Our model included a generic type of L-type channel implicated in pacemaking [49]. In figures 10 A and B, we have used this L-type model to predict the kinetics of the CaAK (*BK+SK*) currents and the inclusion of only this L-type 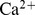 channel can predict the kinetics of the non-inactivating BK (***I*** 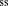) and SK currents well. Inclusion of additional HVA 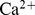 channels improves this model only marginally (not shown). However, in order to model the kinetics of the inactivating BK current (***I*** 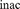), we needed to include the inactivating T-type LVA 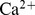 channel observed in SN DA neurons [1], [50]–[52]. We have assumed that there are at least two pools of BK channels. In the first pool, BK channels would be clustered near T-type 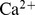 channels because these channels inactivate rapidly which would account for the inactivation of the BK channels. A second pool of BK channels is assumed to be clustered around Ca-HVA channels because the kinetics of L-type 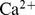 channels can generate 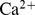 profiles that predict the kinetics of the non-inactivating BK current (***I*** 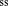) (Fig. 10A&B). In the intracellular space surrounding the first pool, 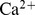 accumulates according to the kinetics of the Ca-LVA T-type channel, and in the intracellular space surrounding the second pool, 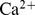 accumulates due to the activation of clusters of Ca-HVA L-type channels. We assume that these types of clusters are different because the 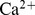 dynamics needed for the generation of BK currents kinetics are different for the ***I*** 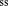 current and for the ***I*** 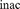 current. In support of this model, a review of the literature indicates that there is ample evidence of the molecular interaction between BK channels and HVA channels [44], [45], but additionally, T-type 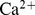 channels and BK channels co-immunoprecipitate in brain tissue [53], and in several neuron types, including cholingergic, thalamic, Purkinje, and choclear, the association of T-type neurons with BK and SK channels controls bursting [32].

**Figure 11:**
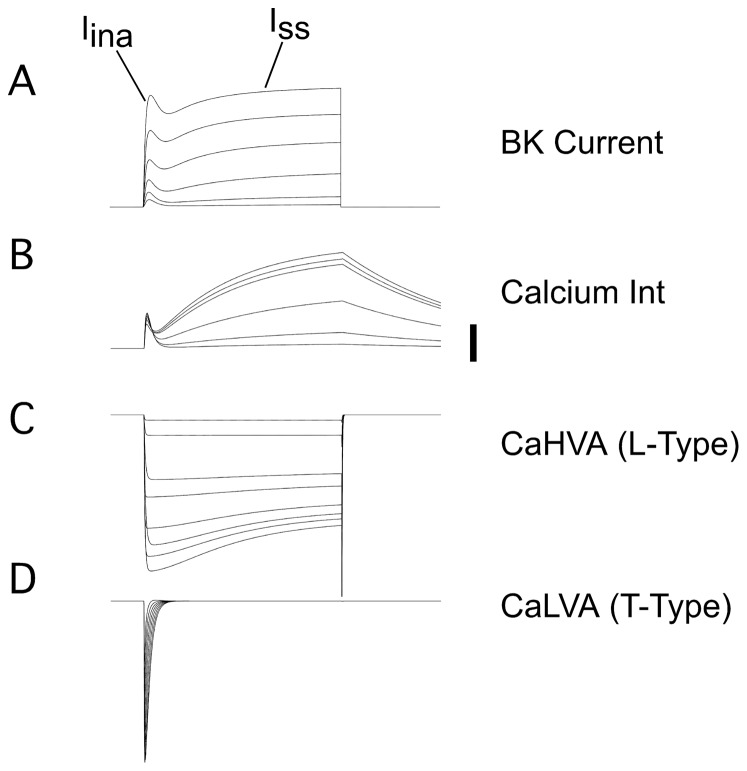
Model of 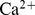 -channel kinetics underlying the BK currents. (A) The BK current was obtained assuming that there are two pools of BK channels as explained in the text. The equations for the activation of BK channels from eqs. (1,2). The equation for the 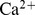 concentration is 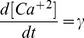 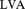 *I* 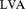
+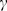 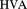 *I* 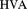 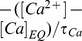. The values for the conductances were changed to give the best fit to the BK kinetics. Typical values for 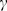 ranged between 0.001 to 0.1 mM s^−1^. (B) 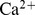 currents derived from the activation of T- and L-Type 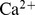 channels generated with the equations above. Bar indicates 20 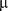
M. (C) Generation of L-Type 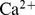 currents. Generalized activation and inactivation forms were used to fit the data for T-type and L-type 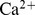 channels [48]–[50] according to the formula 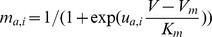, where m can be activation or inactivation(a,i), and time constants were modeled according to the equation 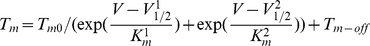. For the L-type Ca channel, 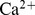 -dependent inactivation was used. (D) T-Type 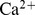 currents were generated with similar equations as in C, except that a voltage-dependent inactivation was used.

Figure 11 summarizes the main features of the model used to predict the kinetics of the BK currents. The top panel shows the BK current kinetics generated by the model. The second panel shows the 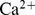 profiles used to generate the BK currents (labeled Ca Int), and the 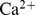 concentrations from the two domains have been added for simplicity of the representation. The third and fourth panels (Labeled CaHVA and CaLVA) show the activation and inactivation functions of the L-type and T-type Ca-currents that we have used for this simulation. These activations generate the Ca-profiles shown in the second panel. For these simulations, we have used a heuristic model with two domains, where the 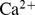 concentrations are de-coupled. In one domain we envision clustered 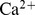 T-type channels and BK channels clustered in a small area. The second domain would consist of a larger area of the cell membrane where clusters of HVA (L-type 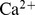 channels) and BK are spread more widely. Because our models so far solve heuristic mathematical equations for the 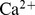 concentration that influences BK channels (Fig. 11 legend), the next step is to see if a more detailed model in which the geometry of the distribution of 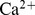 -channels and BK channel is taken into account can reproduce the kinetics of the 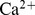 intracellular accumulation for both domains, and can validate the assumptions that L-type and BK clusters are spread over a larger area, whereas the T-type and BK clusters are concentrated in a smaller area. We do this in the next section, where we study if a more realistic model of clusters of 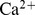 and BK channels can give rise to 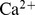 dynamics that are necessary for reproducing both components of the BK current shown in Figures 7,10, &11.

### More realistic model of calcium entry, diffusion and buffering

**Figure 12:**
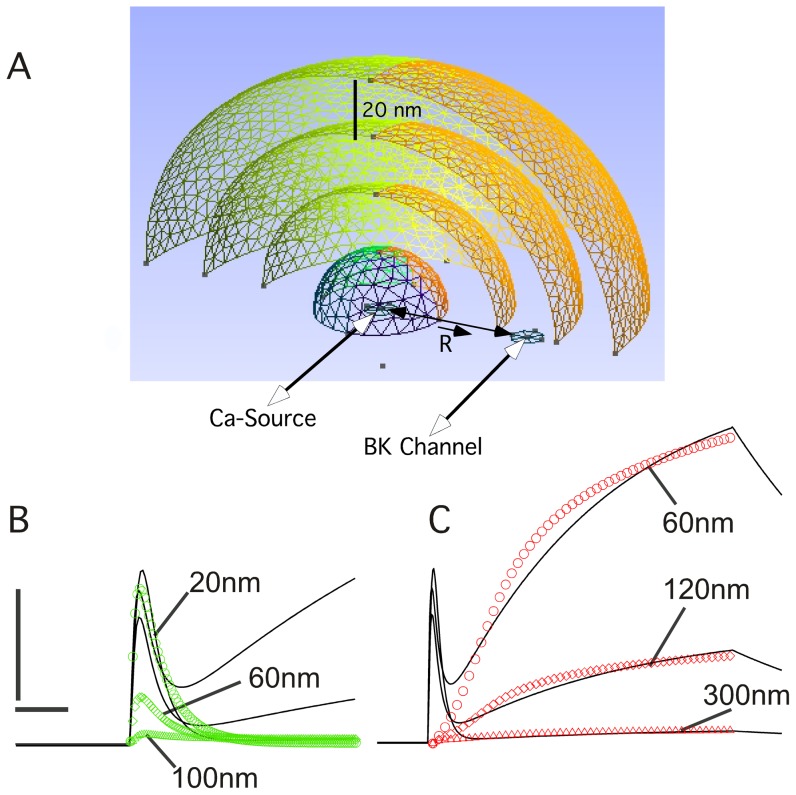
Shell model for solution of 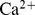 -diffusion problem. (A) The space is divided in spherical shells of thickness of 20 nm and the 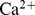 channel is localized at the center of this configuration (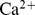 Source). The distance of the BK channel to the 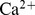 source is indicated by R. The equations to be solved on this geometry are the diffusion equation and the interaction with the mobile 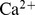 buffer equations 5–7. The mobile buffers Bi are also subject to diffusion. We integrated these equations over the spheres taking N points at the middle of the shells where, 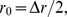 and 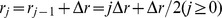. Integrating gives 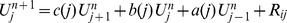, where 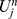 is the calcium concentration at shell j and time n, and similar equations for B_j_, the buffer concentrations for each shell. The coefficients are,
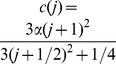
,
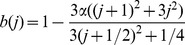
 and 
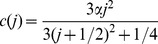
 and 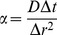. The 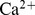 diffusion coefficient is 250 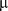
m^2 ^s^−1^ [57]. The buffer diffusion constant and rate constants were taken from ref [57] for calmodulin. **B**. We have solved the matrix equation as explained in the text. The current was converted to Molar s^−1^ using the equation 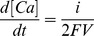, where F is Faraday's constant, i is the current and V is the volume of the shell. The 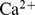 source was assumed to open at time 0 and decay with kinetics similar to the T-type channel. (B) Panel B shows the results of the simulation for shells 1(20 nm, open green circles), 3 (60 nm, open green triangles) and 5 (100 nm, open green diamonds). The black traces show the heuristic 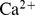 kinetics used in figure 11 to fit the ***I*** 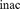 component of BK currents. The bars represent 20 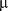
M and 20 ms. (C) Same as in B, except that the 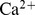 source was distributed over the first three shells were updated according to the rule 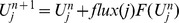 where the flux F was 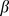 (Shell 1), 0.51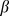 (Shell 2) and 0.36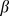 (Shell 3) with 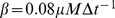. The function F is a saturable function of calcium concentration that represents 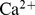 -dependent inactivation. Panel C shows the results for shells 3 (60 nm, open green circles), 6 (120 nm, open green diamonds), and 15 (300 nm, open red triangles). The black traces show the heuristic 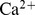 kinetics used in figure 11 to fit the ***I*** 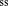 component of BK currents. The bars represent 20 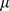
M and 60 ms.

Perhaps the biggest challenge to a realistic simulation of 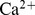 -dependent excitability processes associated with activation and inactivation of channels in the membrane, is the calculation of the concentration of 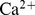 in local domains [54], [55]. Theoretical calculations and numerical simulations of the 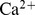 concentration changes from a source of 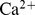 ions, showed that a 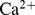 domain of the order of a few tenths of a micron developed within microseconds of the opening of a 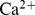 channel [54], [56], [57]. In cells with a high density of channels, these local 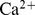 domains overlapped, forming a shell near the plasma membrane [54]. The symmetry of this shell can then be used to calculate the 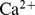 concentration for concentric shell compartments in which the interior of the cell is divided. This approximation requires that the density of 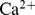 -channels in the membrane be of the order of 100/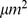 [54]. This density of 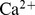 channels is too high for most CNS neurons. The upper estimate of the density of channels can be calculated from the 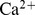 currents observed in SN DA under physiological conditions. This upper estimate of the 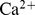 -channel density is 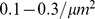, assuming a total current of 100–300 pA for each 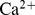 channel type, which we seldom observe under physiological conditions. Even if the current were 10 times higher, the density is still 100 times less than is required for the shell approximation to be valid. Sherman, Keizer, and Rinzel addressed this issue by proposing the existence of high 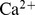 domains around 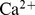 channels [54]. Following this general approach, we have used the local domain mesh to calculate the 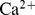 entry from a single source such as a 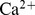 channel (Figure 12A). We want to contrast the 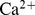 kinetics of single source models with the heuristic models that we have used to simulate BK channel kinetics. We have chosen a mesh size that is big enough so that we can use the elecrophysiological data on 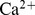 channel kinetics and still use a transport diffusion equation. Transport equations in statistical mechanics require the averaging over sufficiently large volumes and times, so that thermodynamic quantities derived from canonical ensemble partition functions make some sense. Smaller meshes are problematic because one has to estimate 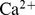 concentrations in very small domains, in which one integrates over very small times and thus the stochastic nature of the channels ought to be taken into account. Reliable kinetic electrophysiological data are only available in the 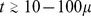
s scale. Following these ideas, we have solved the diffusion equation using the geometry shown in figure 12A,
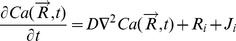
(5)and,
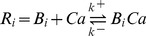
(6)represent the 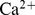 diffusion and binding to buffer B_i_ (Buffer was assumed to diffuse also). These equations were solved with the geometry of figure 12. The J_i_ represents the flux of 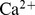 into the cell through 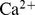 channels and 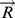 is the vector from the center of the geometry to a point (x,y,z). We have integrated over the shells and used Gauss theorem to simplify the diffusion equation. The results of the integration give
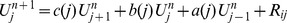
(7)and 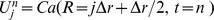 where U is the 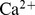 concentration in the middle of the j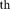 shell, 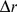 is the distance between shells, and the coefficients a, b, and c are shown in the legend of figure 12; R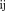 represent the chemical equations for Ca-binding to buffer B_i_ and similar equations for diffusion of buffers. Equation 7 was solved with a tridiagonal matrix decomposition into an upper and lower diagonal component such that for the equation 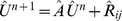, the matrix 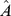 can be decomposed as 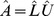 where 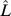 and 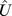 represent lower and upper diagonal decomposition matrices respectively. These equations were solved using Octave (a GNU-LINUX free version of MATLAB, Figure 12). The results can be seen in figure 12 A, B, and C. For the first pool of T-type 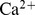 -channels and BK channel clusters (pool1), we use a 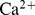 channel at the center. For the second pool of L-type 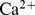 -channels and BK channels, we use a distribution of 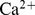 channels represented by an exponential distribution with a characteristic length scale (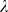), such that distribution of 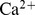 channels at a distance x from the source decays for 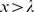, as a function of x is 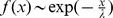.

In figure 12B, we see the 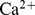 concentration for a single 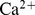 channel source as a function of time and parametrically for distance. In this case, the calcium channel is located at the center of the shell and the figure shows the evolution of the intracellular 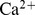 as a function of the distance. Open green circles labeled 20 nm show the time evolution of the 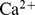 concentration at the first shell, which is 20 nm (200 Å) in size (labeled 20 nm). The green diamonds show the 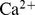 concentration in the third shell at a distance of 60 nm from the center (labeled 60 nm). Finally, the green triangles show the 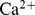 concentration of the fifth shell, 100 nm away from the center where the 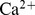 channel is located. We assume that the 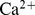 channel at the center is inactivating with kinetics similar to those of the T-type 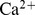 channel shown in figure 11. Superimposed on this figure are the heuristic 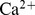 kinetics used in figure 11 to generate the BK current kinetics. In figure 12B, the 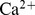 dynamics of the first shell fit well the heuristic model of the inactivating 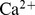 dynamics used to simulate the ***I*** 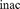 BK current. This model would account well for the inactivating BK current kinetics.

Figure 12C shows the results obtained with the spatial configuration for pool 2 consisting of L-type 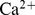 channels and BK channel clusters. In this case, a larger cluster with a space scale of 60 nm (red circles, fig. 12B) is used to produce 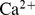 currents that were used to generate the 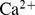 kinetics of figure 11, used to reproduce the kinetics of the non-inactivating ***I*** 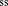 BK current (Fig. 12C, black traces). Figure 12C also shows the simulations when the 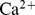 channels are distributed over larger scales (red diamonds l = 120 nm and red triangles, l = 300 nm). From these simulations it is clear that a larger number of 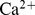 channels are required to be spread over larger areas in order to generate the kinetics necessary to reproduce the non-inactivating BK currents, and that distributing the 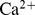 channels over larger areas attenuates the 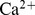 current rapidly. Based on the results of these simulations, we propose that inactivating BK currents require smaller clusters of BK and 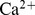 channels (T-type) that can hyperpolarize a small patch of membrane whereas the non-inactivating BK currents require more widespread clusters of BK and 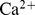 channels(L-type), and that these larger density of Ca-channels and BK currents have the ability to hyperpolarize larger membrane areas.

## Discussion

We have examined the developmental changes in excitation, during a critical period of development of the nigro-striatal DA projection. In embryogenesis, cells that give origin to SN and VTA neurons arise from the ventricular zone of the aqueduct of Sylvius, and traverse in radial tracts to the ventral mesencephalon (Figure 1A) [58], [59]. The first dopamine neurons appear in the rat ventral diencephalon at E13, and the first DA fibers reach the striatal anlage around E14 [38]. On E19, the first signs of the compartmental organization of the nigral DA projection on the striatum appear, and during the first three weeks of postnatal development the nigral innervation of the striatum undergoes a process of extension, refinement, and maturation [38]. The developmental up-regulation of the outward current coincides with the time in which DA neurons are extending their axonal arbors and making new synapses with striatal neurons [38]. It is possible that presynaptic SN DA axons interact with postsynaptic striatal neurons, and that this interaction could generate a retrograde signals that would cause the upregulation of CaAK reported in this paper. In cilliary ganglia, it has been shown [60] that the developmental regulation of BK channels depends on preganglionic contacts that provide 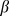-Neuregulin-1 (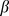-NRG-1) and target contacts that provide TG 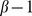. In the midbrain, total BK mRNA is upregulated and this upregulation is splice-variant specific [61]. In glioma cells, BK channels functional upregulation was shown to be a downstream target of ErB2-NRG signaling [62] and disruption of this signaling mechanism resulted in substantial decreases of functional BK channels on the cell surface. Dopamine neurons express the high affinity NRG1 receptor Erb4, which is developmentally regulated [63] and NRG1 is expressed in the striatum [64] and it is also developmentally regulated (J. Ramirez, unpublished observations). The interaction between the NRG1 ligand and its ErbB receptor could occur during development when SN DA terminals invade the striatal anlage. Recently, it has been found that injection of NRG1 in the dorsal vicinity of the SN causes a dopamine overflow in the striatum [65] and systemic injection of a portion of the extracellular NRG1 domain-known to cross the blood-brain barrier- protects dopaminergic neurons in a mouse model of Parkinson's disease and produces an increase of striatal DA in a matter of days [66]. These effects could be explained by a modulation of excitability caused by the interaction of NRG1-ErbB on SN DA neurons similar to the proposed increase in expression of CaAK channels in development proposed here.

Future research will concentrate on the study of the signaling mechanisms that are responsible for the upregulation of CaAK channels in DA neurons and this line of research provides an avenue to study the influence of striatal, subthalmic, or pontine innervation on the development of excitability in dopamine neurons.

It is interesting to speculate that if CaAK could also participate in the regulation of dopamine release by DA neurons of the ventral tegmental area (VTA)–neurons that share a common developmental origin with SN DA neurons– then CaAK channels could be important for the understanding of the abnormal DA release in the ventral striatal anlage, which is involved in schizophrenia. In support of this hypothesis is the observation that several CaAK regulators are known to have antipsychotic properties, including diazoxide, a commonly used neuroleptic [67] and that neuregulin has been identified as one of the genes involved in the susceptibility to schizophrenia [68].

### The outward 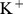 current early in development: implications for excitability and DA release

The first component of the outward current examined, the ***I_A_*** subclass, is not upregulated in the first two weeks of postnatal development, while the ***I*** 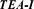 and ***I*** 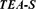 subclasses of the outward current are up-regulated considerably in this period. The simple kinetics of the ***I*** 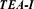 subclass, and its invariance with development suggest that the channels underlying this subclass constitutes a relatively homogeneous functional unit. SK channels that are voltage insensitive have been reported to be expressed in the SNc [66] and are likely part of this component because the kinetics are similar to those reported in the literature and are sensitive to apamine (not shown). A large proportion of the ***I*** 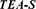 sensitive current can be blocked with BK channel toxins IbTx and CbTx, showing that BK channels are an important part of this current. The ***I*** 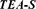 currents are nearly absent at P0, producing a non-inactivating outward which is not modulated by extracellular 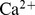 and is insensitive to TEA, CbTx and IbTx. Single BK channels have been reported in SN DA neurons [34] and we observed them readily, often expressed several in one patch (Fig. 9 C,D). We measured the half-activation point of the activation function for BK channels (Fig. 9E) as a function of both voltage and calcium and based on these experiments, we constructed a model to see if we could predict reasonably well the outward current observed in real neurons. The results show close agreement with our model (Fig. 10 A,B).

At P0, only the ***I_A_*** current and the slow (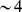 ms) TEA insensitive (***I*** 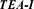) current contribute to the outward current. These results have implications for the form of the AP fired by SN DA neurons early in development. During the first days of postnatal development, three main factors contribute to the large width of the action potential in SN DA neurons. The first factor is the depolarized resting potential of the young neurons that inactivates nearly all the ***I_A_*** current. This ***I_A_*** inactivation reduces the number of open 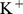 channels that can be recruited during the repolarization phase of the AP, slowing down the AP. The second factor that influences the width of the AP is the absence of the ***I*** 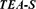. The lack of ***I*** 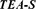 contributes to the increase in the width of the action potential as well, because the ***I*** 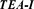 current has slow kinetics of activation of the order of 4–5 ms. A third factor that contributes to the large width of the AP is the small amplitude of the ***I*** 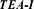 current present during the first few days of postnatal development. These three factors contribute to the immature shape of the AP and the absence of AHPs observed early in early postnatal development.

### The developmental regulation of the *I* 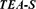 and *I* 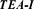 currents

The ***I*** 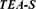 current develops rapidly after P0 while the resting potential becomes hyperpolarized. During the first few days of postnatal development, the outward current becomes sensitive to TEA, CbTx, and IbTx. The TEA sensitive currents (***I*** 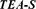) activate and inactivate with complex kinetics and they can influence the shape of the AP, because the ***I*** 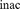 component activates rapidly. The BK channels that contribute to early frequency adaptation in neurons are fast inactivating so that they can repolarize the cell rapidly, without causing excessive delay in the trigger of the next action potential, which would happen if the inactivation were lost [44], [69]. This early frequency adaptation is achieved by the activation of fast inactivating BK channels. In CA1 pyramidal neurons, it has been found that this BK current has to inactivate substantially during the duration of one action potential [69].

Furthermore, as development progresses, both the hyperpolarization of the neurons and the increase in the ***I*** 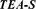 current contribute to reduce the width of the action potential, an opposite effect of the early depolarization and absence of ***I*** 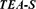 which widened it during the first week of postnatal development. The hyperpolarization of the neurons relieves the voltage-dependent ***I_A_*** inactivation, while the influence of ***I*** 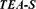 increases the rapidity with which the AP can return to the resting potential. The increased TEA and BK toxin sensitivity of the 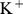 outward current show that the participation of BK channels is a developmental addition, perhaps critically important to the process of integration of voltage and 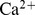 signals at the soma. The integrating ability of BK channels is likely to be important for movement regulation, because SN DA neurons respond with bursts volleys to movement related cues [70] indicating an adaptation mechanisms that can be provided by BK channels [33]. These adaptation mechanisms could be further enhanced by the presence of, and the co-localization with 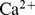 -entry sites, such as 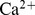 channels or ionotropic excitatory receptors such as NMDA and neuronal nicotinic receptors (nAChR), both of which are found on SN DA neurons [23], [26].

### Clusters of BK and 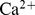 channels and their function

In our model, the characteristic separation between T-type 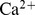 channels and the BK channel is of the order of 20 nm which is consistent with molecular contact. On the other hand, while the separation between L-type and BK channels could also be consistent with molecular contact, the delayed opening of the 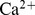 source required to achieve high 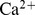 concentrations, suggests to us that that the L-type-BK clusters are more widespread over a larger area of the membrane such that effects of spacial distribution, voltage dependence, and 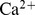 diffusion to neighboring clusters are more important than in the T-type-BK clusters. Indeed, the results of our modeling support this conclusion (Fig. 12 B,C). Also, the total charge moved by the BK-L-type is much larger than the BK-T-type clusters, so that the charging of the membrane can influence the voltage over larger areas. Our model of BK- 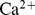 channel clusters explains quite well the kinetics of the inactivating as well as the non-inactivating BK currents. We propose that the two types of BK- 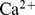 channels clusters have two different functions. The fact that the channels acting locally inactivate rapidly indicate to us that their action may function to hyperpolarize localized pieces of membrane fast but transiently, acting like coincidence detectors that work synergistically to reinforce two or more localized domains to incoming dendritic signals, thus adjusting the strength of coincident synaptic inputs into SN DA neurons. How would this mechanism work? We propose that due to the difference in voltage-dependent activation of T-type 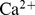 channels, a small depolarization, typical for example of a synaptic epsp, could in fact activate the T-channel which would tend to enhance the epsp; this enhancement would be followed by a hyperpolarization caused by the activation of the local BK channels that surround the T-type 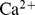 -channel activated; at the same time that the BK channels are being opened, the 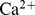 channels are being inactivated (See Figure 12). This sequence of events produces at first a depolarizing signal that reinforces the input(by the opening of T-type Ca channels), followed by a transient hyperpolarization of the BK channels and the inactivation of the T-type 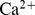 channels. With this mechanism, two incoming depolarizing signals arriving at precisely the same time at the soma, could be summed and enhanced, whereas two epsps arriving at slightly different times would be suppressed or diminished by the hyperpolarization of BK channels and the inactivation of the T-type channel. This is a plausible mechanism that would work as a coincidence detector; and in addition to this, it would be a non-linear summation dependent on neighboring 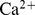 domains, due to the nonlinear character of BK and 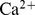 channel activation and inactivation. This nonlinear summation may amplify certain types of signals over others. Consistent with this proposal is that inactivating BK currents in our experiments activate at lower voltages, a feature that is replicated by our model (Figures 7 and 11) and that the 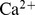 concentration is fast and transient, and these two features are necessary to replicate the kinetics of the inactivating BK current. Finally, it is also possible that these precise timing mechanisms are important for the generation of bursts that are apparently important for the cognitive effects in the function of SN DA neurons, such as prediction of value [20]. The dendritic 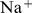 currents that one observes in SN DA neurons could participate in the reinforcement of dendritic signals, however blocking these dendritic 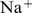 channels with TTX does not seem to alter bursting [17], although in this paper the bursting is caused by a rather large application of glutamate to the dendrites, and it is possible that functional bursting produced by much smaller synaptic currents could be substantially different from the bursting induced here [17].

What are the probable functional implications of the lack of excitability in the somatic compartment of SN DA neuron? It is possible that in immature SN DA neurons, the role of somatic excitability is limited. One possible reason for this is that in the brain, excitatory and inhibitory input are not mature in early postnatal development [71]. In many areas of the rat brain, including the SN, synaptogenesis develops slowly and gradually from E20 to a peak around P20 [71]. We speculate that the electrical role of the soma as an integrating synaptic input develops in parallel with the synaptic input. In this case, a specific dendro-axonal location might serve as an initiator of AP, bypassing the soma, in the cases where the axon emerges directly from the dendrite [17]. In other cases, the dendritic signals might traverse the soma, which could behave as a passive element-toward the site of initiation. Later in development, with the maturation of excitatory input from the PPN and the STN [22], [23] and the inhibitory input from GP and SNr [12], the somatic compartment could assume the role of integration of excitatory and inhibitory inputs. Recently, a study found that the mechanism of AP initiation was more conventional than originally thought and the site of AP initiation seemed to be close to soma-axonal areas independently of whether the AP was part of a burst or spontaneously recorded [18].

The components of the outward current play an important role in the regulation of excitability and the generations of electrical patterns necessary for the appropriate release of DA in the striatum. Models of the electrical activity can use our results for a better understanding of the electrical properties of SN DA neurons which include the variability in AP as well as the different firing modes exhibited by these neurons. These results are important for understanding the role that the SN DA neurons play in modulation of the basal ganglia.

We propose that given the state of electrical immaturity of SN DA neurons, the mechanism of action selection is in a locked state that cannot regulate the transformation of stimuli into effective connections especially at the level of the spiny medium neurons in the striatum, one of the main site of regulatory actions of dopamine. This locked state would prevent dopamine neurons from reinforcing cortico-striatal synapses that do not have a functional experiential based value early in development. It is known that early in development the action of psychostimulants such as amphetamine has a paradoxical effect on the firing rate of dopamine neurons [72] and this effect could be interesting evidence of the changes of excitability and its regulation by dopamine in the transformation from inactive to active processing of action selection regulated by dopamine. Recent experiments in mice show that 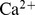 -currents are upregulated in DA neurons in culture [73] and this observation together with our reported findings suggests a state of rapid changes in excitability in SN DA neurons indicating that these neurons and perhaps all DA neurons in the midbrain undergo a process where they transition from a non-functional immature state to a functional mature one in the first month of postnatal life. Given all of these findings, it would be interesting to examine the effects of dopamine on the synaptic plasticity mechanisms at cortico-striatal synapses early in development. One important question that immediately arises is when does dopamine start modulating cortico- striatal synapses and whether this modulation changes as development proceeds. There is some controversy in the literature as to whether the modulation of dopamine produces bidirectional or unidirectional modulation of these synapses in mature animals [6], and the examination of this issue early in development may help understand this complex phenomena.

Finally, it is interesting to note that our results would explain why cattle get sick when eating infected grass with an endophyte fungus containing lolitrem B, a powerful BK channels blocker [74]. Sheep, cattle and horses can all become affected and show tremors, limb rigidity and ataxia. The authors attributed these phenotypes to the expression of Cerebellar BK channels. As suggested in the present paper, CaAK channels (BK and SK) play an important role in the regulation of excitability in SN DA neurons, and thus their influence in the regulation of movement should be evident when either of these channels are blocked as it is seen in the Ryegrass Staggers [74] or in alcohol intoxication known to block SK and block or stimulate BK channels [75], producing a remarkable-and for some, no doubt, enjoyable- incoordination. Our BK results would predict that the blocking of BK currents in SN DA neurons should have a disabling quality which would closely resemble that observed in Parkinson's: tremors, incoordination, and the inability to initiate movement. These movement phenotype has to be considered in addition to the ataxic phenotype which is typical of cerebellar dysfunction.

## Materials and Methods

### Slice preparation

All the experiments were carried out using protocols approved by Temple University Institutional Animal Care and Use Committee (Temple University IACUC), CUNY IACUC (College of Staten Island) or the Comité de Etica (Ethics Committee) at UROSARIO. Sprague-Dawley rats were injected (IP) with sodium pentobarbital (40 mg kg-1). After anesthesia, animals were decapitated and their brains quickly removed and placed in an oxygenated Mid-Sucrose solution (in mM, 227 sucrose, 26 NaHCO3, 2 KCl, 1.5 NaH2PO4, pH 7.4 with 95%O2, 5%CO2. Brain slices were cut in sections 250–400 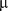
m thick, in the frontal plane with a vibrotome (752 M Vibroslicer, Campden Ins. UK). Slices were placed in oxygenated ACSF (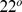
C) for at least one hour before the recording. Slices remained viable for several hours. Electrophysiological recordings were carried out at room temperature (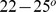
C). CbTx, IbTx, and apamin were purchased from RBI.

### Immunocytochemistry

Dopamine neurons were labeled with monoclonal mouse anti-tyrosine hydroxylase antibody (RBI, Cat-186). Secondary antibody was either a rhodamine-conjugated goat anti-mouse IgG (Rockland, 610-100-121), or a biotin-SP-conjugated donkey antimouse IgG (H+L) (Jackson Immuno Research, 715-065-150). The Vectastain ABC Elite Kit was used for the avidin-biotin reactions, using an HRP-avidin system. Fluorescein-conjugated to avidin (Vector) was used to label biotin filled cells. DAB tablets (Sigma) were used as a substrate for the peroxidase system. Slices were fixed in 4% paraformaldehyde in ACSF, blocked with 10% NGS and 0.05% TRITON in ACSF or in PBS. Endogenous peroxidase was inactivated with 0.3% H202 in PBS for 15 min. After wash, the primary antibody was incubated (1∶1000–5000) in PBS overnight at 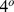
C, washed 3 times, and incubated in secondary antibody overnight at 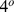
C. Rhodamine fluorescence was observed in a Leika TCS NT confocal microscope, with a 568 nm excitation laser or in an Olympus BX60.

### Electrical recording

Perforated patches were used as reported [76] with the following modifications. Pipette solution was prepared by dissolving 3 mg of LuciferYellow (LFY) (Sigma) and 1 mg of Biocytin (Sigma) in 10 ml of intracellular solution. Gramicidin was used to back fill electrode at a concentration of 0.14 mg/ml and was sonicated for 10 min before use. Pipette solutions (ICSF) consisted of (mM), 120 K-gluconate, 10 HEPES, 0.2 EGTA, 10 KCl, 10 NaCl, 2 MgCl2, pH7.4. For single channel recordings, the 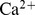 concentrations were prepared using two solutions containing (mM) 120 K+Gluconate, 10 HEPES, 10 KCl, 10 NaCl, 2.0 MgCl+4EGTA(Sol.1) or 4 mMCaCl_2_ (Sol. 2), pH 7.4 according to the methods of Bers [77], [78]. Sols. 1 & 2 were mixed at different dilutions. Calcium Green fluorescence was measured according to Molecular Probes Ca-Buffer calibration kits (Calcium Green-1,2 and Ca-Calibration kits, Molecular Probes) and compared to the fluorescence obtained in the mixing of solutions 1 & 2, to achieve approximate 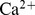 concentrations of 0.1, 1 and 10 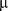
M. For single BK channel recordings, the Po was calculated with an all point histogram [79]. For these recordings the bandwidth filter was set at 10 Khz and the digitization rate is at 100 khz. Only patches with one channel were used to measure Po and this was determined by increasing the Ca concentration and voltage so that there are no multiple openings. The toxin sensitivity was measured in 3 outside-out patches by application of high concentration 1 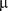
M of IbTx and visual inspection of the record showed few channel openings after the application of the toxin.

After establishing a gigaseal, we waited for up to 40 minutes for the access resistance to reach acceptable levels. In a typical recording, the access resistance developed over the course of 20–30 minutes (Fig. 2A). Liquid junction potentials were carefully adjusted at the beginning of the insertion of the electrode in the extra-cellular fluid, and immediately after establishing the seal. Typical corrections were 2 mV. Electrodes were pulled from soft glass (Corning #8161) to produce a fat short shank with a typical resistance of 2–3 M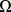. Electrodes were coated with Sigmacote (Sigma). Chloride pellets were fabricated in house, coating the Ag wire using melted AgCl powder in a crucible. We selected recordings with access resistance (Ra) in the range of 3–20 M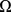, but most recordings were made with access resistance in the 6–9 M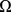 range and under these conditions we measured the typical response time of the 200B amplifier to be 300–500 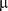
s, in agreement with previous published results [80]. Breaking the membrane by suction often improved the access resistance, but at the cost of severely degrading the current by rundown. At the end of the experiment, we broke the patch mechanically to allow access of LFY and biocytin into the neuron to label the cells. The whole-cell capacitance current did not follow a single exponential decay, and could be fit with a double exponential decay. For P0, 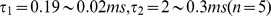. We calculated the membrane capacitance to be 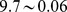 pF for P0 (n = 5) and 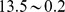 pF for P7 (n = 4). These values qualitatively agree with those reported previously [50], and indicate a modest increase (1.39) in the somatic membrane, which can be hinted by the slightly larger shapes of the neurons as they develop (Fig. 1C). Axon 200B, and 200A amplifiers were used for the experiments. Software acquisition programs were HEKA Pulse and PulseFit in a PC ADM processor, running an INSTRUTECH ITC-18 Board, with additional routines written in Microsoft C++ Visual5.0. An Olympus BX50WI with infrared optics and swing out objectives was used for all the recordings.

Double Labeling. Double-labeled neurons were obtained as follows. After recording and fixation for 1 hr, the neurons were viewed and their LFY fluorescence recorded. After incubation with the 1o antibody, neurons were incubated in fluorescein-avidin. The fluorescein picture is then compared with the original LFY. The HRP DAB reaction is made in the microscope so that the peroxidase image can be superimposed on the fluorescent image as it develops. This helps with identification of DA recorded neurons in areas of high density of TH positive neurons.
